# Ambulance Attendance in the State of Queensland, Australia: Exploring the Impacts of Heatwaves Using a Retrospective Population-Based Study

**DOI:** 10.1017/S1049023X25101192

**Published:** 2025-06

**Authors:** Jemma C. King, Hannah M. Mason, Amy E. Peden, Gerard Fitzgerald, John Nairn, Nicole Mandalios, Kerrianne Watt, Emma L. Bosley, Richard C. Franklin

**Affiliations:** 1. Discipline of Public Health and Tropical Medicine, College of Public Health, Medical and Veterinary Sciences, James Cook University, Townsville, Queensland, Australia; 2. School of Population Health, Faculty of Medicine and Health, University of New South Wales, Sydney, New South Wales, Australia; 3. School of Public Health and Social Work, Faculty of Health, Queensland University of Technology, Brisbane, Queensland, Australia; 4. School of Public Health, University of Adelaide, Adelaide, South Australia, Australia; 5. Disaster Management Branch, Queensland Health, Brisbane, Queensland, Australia; 6. Information Support, Research & Evaluation, Queensland Ambulance Service, Brisbane, Queensland, Australia; 7. School of Clinical Sciences, Faculty of Health, Queensland University of Technology, Brisbane, Queensland, Australia

**Keywords:** ambulance, heatwaves, prehospital, Queensland, transport

## Abstract

**Objective:**

This study explores the impact of heatwaves on emergency calls for assistance resulting in service attendance in the Australian state of Queensland for the period from January 1, 2010 through December 31, 2019. The study uses data from the Queensland Ambulance Service (QAS), a state-wide prehospital health system for emergency health care.

**Methods:**

A retrospective case series using de-identified data from QAS explored spatial and demographic characteristics of patients attended by ambulance and the reason for attendance. All individuals for which there was an emergency call to “000” that resulted in ambulance attendance in Queensland across the ten years were captured. Demand for ambulance services during heatwave and non-heatwave periods were compared. Incidence rate ratio (IRR) and 95% confidence intervals (CI) were constructed exploring ambulance usage patterns during heatwaves and by rurality, climate zone, age groups, sex, and reasons for attendance.

**Results:**

Compared with non-heatwave days, ambulance attendance across Queensland increased by 9.3% during heatwave days. The impact of heatwaves on ambulance demand differed by climate zone (high humidity summer with warm winter; hot dry summer with warm winter; warm humid summer with mild winter). Attendances related to heat exposure, dehydration, alcohol/drug use, and sepsis increased substantially during heatwaves.

**Conclusion:**

Heatwaves are a driver of increased ambulance demand in Queensland. The data raise questions about climatic conditions and heat tolerance, and how future cascading and compounding heat disasters may influence work practices and demands on the ambulance service. Understanding the implications of heatwaves in the prehospital setting is important to inform community, service, and system preparedness.

## Background

Heatwaves are significant meteorological events that impact the health of humans, animals, and ecosystems.^1^ Globally, between 1950 and 2011, the intensity, duration, and number of heatwave days have increased.^2,3^ The Intergovernmental Panel on Climate Change (IPCC; Geneva, Switzerland) projects heatwaves will increase over most land areas internationally, including in Australia.^3^ These climatic events occur across multiple days. There are differing definitions for what constitutes a heatwave, but all heatwave classification systems acknowledge that heatwaves are characterized by an increase in temperature and the stability of this extreme temperature across time.^4^

The anticipated increased frequency and intensity of heatwaves necessitates an exploration of how heatwave events influence human health and health service usage. A deeper understanding of these effects, both now and into the future, can inform health system strengthening and other mitigation approaches.^4,5^ A global review by Campbell, et al indicated that tropical climates are notably under-represented in the literature.^6^ Queensland, an Australian state partially located within the tropics on the eastern seaboard of the country, has a decentralized population with significant regional centers. This decentralization can compound and amplify heat health impacts in prehospital settings.

Heatwaves are noted to influence human physiology, cognition, and mood, mediated by hydration, medication, and pre-existing medical conditions.^4,7^ The escalation of these heat health impacts can be further compounded by the presence of humidity, which is noted to be a factor in the lethality of heatwaves.^8^ It is via these direct and indirect human health effects that heatwaves influence health service usage. A multi-national review and meta-analysis conducted by Xu and colleagues in 2023 determined a seven percent increase in all-cause ambulance dispatches for each 5°C increase in mean temperature.^9^ In addition, a recent review of the Australian literature by Mason, et al explored the impact of heatwaves on health system demand and found that ambulance demand increased, particularly for cardiovascular, respiratory, nervous system, and mental and behavioral conditions.^4^

It was recently demonstrated that emergency calls to Queensland Ambulance Service (QAS; Brisbane, Queensland, Australia) increased by 12.7% during heatwaves.^10^ However, there has been limited exploration of how heatwaves impact prehospital and retrieval services in rural and remote areas and whether there are differences in demand between climate zones.^11,12^ This is increasingly important given recent data highlighting that humidity and wet-bulb temperatures are already in the upper physiological tolerance limits in many tropical and sub-tropical locations around the world, suggesting tolerance limits even with acclimation will be challenged by heatwave events.^13^

The primary aim of this study is to explore the impact of heatwaves on ambulance attendance in Queensland. The secondary aim is to explore how this may vary by demographic characteristics, geographical remoteness, climatic conditions, and reason for attendance.

## Methods

### Setting

Queensland is the second largest state in Australia with a population of over five million and covering a land area of 1,727,000 square kilometres.^14,15^ The QAS is a state-wide health service organization that provides emergency response from 302 ambulance response locations organized into eight geographical regions and 16 districts that align with the hospital and health service boundaries (note: during the study period, there were 15 districts).^16^ The QAS is the fourth largest ambulance service in the world and provides several emergency and non-emergency services such as prehospital Emergency Medical Services (EMS), transport services, and the coordination of aeromedical services (via Retrieval Services Queensland; Kedron, Queensland, Australia).^17,18^ This offers the opportunity to explore heatwave-associated demand across the entire state to help understand if there are differential impacts that can inform health service planning.

Since 2003, ambulance services have been free for Queensland residents to use within the state; this differs from many other states and territories in Australia.^19^ While there are still some exceptions in Queensland for no-cost usage amongst non-Queensland residents, the absence of a financial impediment to demand allows the examination of a direct association between environmental conditions and other factors on demand.

In Queensland, ambulances are accessed by calling the national emergency services number (000) and asking for the ambulance service. Ambulance call-takers determine the reason the service is needed and other pertinent details, such as location, to prioritize and determine the nature of the response required using the Medical Priority Dispatch System (MPDS).^10^ The appropriate resources are then dispatched. Typically, approximately 80% of QAS attendances are transported away from the scene (ie, to the hospital).^20^ The reasons for non-transport can include that appropriate care is provided on scene, the paramedic determines the individual does not need to go to a hospital, or the patient refuses transport.

Data on all events attended by QAS from January 1, 2010 through December 31, 2019 were extracted and de-duplicated so that there was only one unique record for each incident.^21^ Information about events that are attended by paramedics is recorded on the ambulance report form. During the study period, ambulance report forms were captured on two different systems: electronic ambulance report form (eARF - January 1, 2008 through December 31, 2017) and digital ambulance report form (dARF - from January 1, 2018). Extracted variables were age and sex of the patient, postcode of attendance location, primary diagnosis (dARF) or final assessment (eARF) - reason for attendance as recorded by attending paramedic, primary complaint (dARF) or patient complaint (eARF) - reason for attendance as described by the patient, MPDS code (as recorded by call-taker), case nature (eARF only - paramedic’s assessment of nature of incident), and cause of injury (dARF only, pre-defined response categories).

A composite variable describing the reason for attendance was created, mostly based on final assessment (eARF) and primary diagnosis (dARF). Reason for attendance was derived from these variables in 88.5% of records. Response categories in primary diagnosis, final assessment, and MPDS were reviewed by the research team, including a medical expert and QAS representatives, to create new categories aligned with the research priorities and best reflected existing categories in the QAS data. Supplementary File 1 (available online only) shows additional information.

### Heatwave Determinations

A heatwave is defined by the Bureau of Meteorology (BoM; Melbourne, Victoria, Australia) as “when the maximum and minimum temperatures are unusually hot over three days. This is compared to the local climate and past weather. It takes more than a high daily maximum temperature to make a heatwave. It’s also about how much it cools down overnight.”^22^ Importantly, for this definition, it is local cumulative excess heat over a sequence of unusually hot days and nights. In this study, Excess Heat Factor (EHF) was utilized to determine heatwave events for the period January 1, 2010 through December 31, 2019. This method was developed by Nairn and Fawcett and adopted by the BoM in Australia.^23^ This metric is a statistical parameterization of heatwave intensity and severity using temperature anomaly data after taking into account the effect of preceding localized temperatures.^23,24^ While some other jurisdictions have previously used localized heatwave categories and triggers, Queensland Health (Brisbane, Queensland, Australia) has used the EHF and the BoM’s heatwave intensity categories as part of its state-wide heat action plans to inform and trigger messaging and responses during heatwaves since 2015.^25^

Data for EHF were supplied by the BoM for the period January 1, 2010 through December 31, 2019. Data were analyzed using the Australian Statistical Geography Standard (ASGS) Statistical Areas Level 2 (SA2), which is a commonly used level of analysis to explore population statistics as it provides detailed economic and social data but at a higher lens of analysis to avoid the need to apply confidentiality restrictions.^26^

### Climatic Conditions

**Figure 1.**
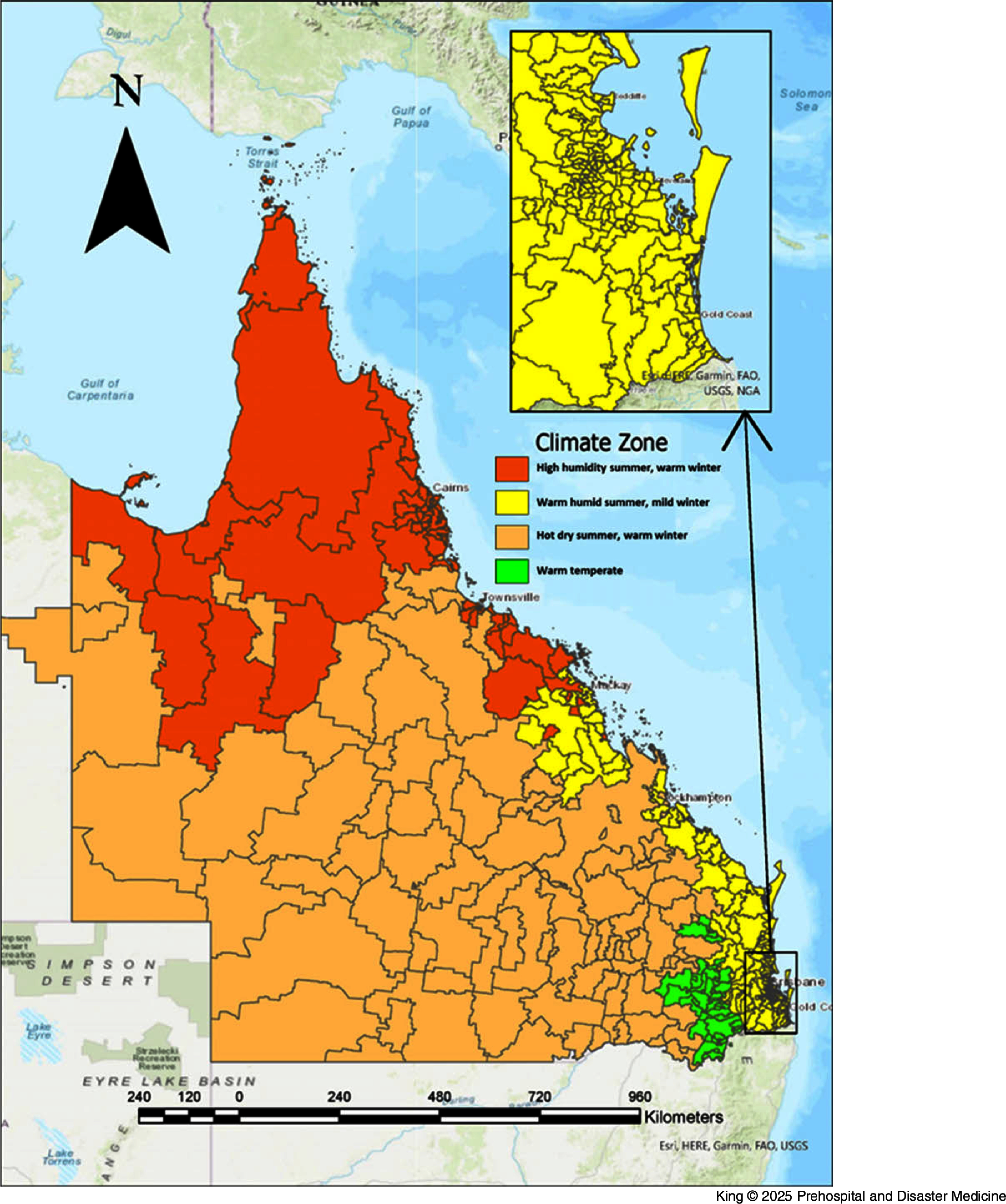
ABCB Climate Zone Map of Queensland, Based on Postcodes. Abbreviation: ABCB, Australian Building Codes Board.

There are five climate zones in Queensland according to the BoM based on temperature and humidity, and there are four climate zones determined by the Australian Building Codes Board (ABCB; Canberra, ACT, Australia) that align with the different heating and cooling requirements within the state.^27,28^ The ABCB zones were created using the BoM climatic data and are used in this paper (Figure 1).^27,28^

### Geographical Data

A postcode (also known as a zip code) is a four digit code that is created and maintained by the national postal service, Australia Post, to assist in mail delivery and align with gazetted suburb/locality boundaries across the country.^29^ The Australian Bureau of Statistics (ABS; Canberra, ACT, Australia) recognizes postcodes are commonly used as a makeshift way to explore geospatial data and have created correspondence files to check concordance from one unit of geographic region to another type.^30^ Postcodes are used in this paper to enable matching of heatwave events, climate zones, and ambulance attendance.

Remoteness data have been constructed using the ASGS remoteness structure classifications correspondence file for postcodes. The remoteness structure has five levels of remoteness with each level reflecting relative road distance access to services; this measure was developed using the Accessibility and Remoteness Index of Australia and enables comparability across locations in Australia.^31^ The five levels of remoteness are major cities, inner regional, outer regional, remote, and very remote.^31^

### Analysis

A three-phase matching approach was utilized. If a specific day in a specific postcode did not have a heatwave day across the ten-year period, then that postcode day was excluded. For example, if there was never a heatwave in Thallon on April 4, this postcode day (Thallon Postcode 4497 for April 4) was excluded. Similarly, if a specific postcode day did not have an ambulance presentation for the ten years, this postcode day was excluded. For example, if there was never an ambulance presentation in Thallon on April 4, this postcode day (4497 for April 4) was excluded. The third level of matching occurred by merging the ambulance and heatwave datasets so that only postcode days that had the potential for heatwaves and ambulance attendances were captured.

For more information about this process, see the detailed overview in Mason, et al (2023).^10^ This matching process enabled comparison of times of the year and locations which have historically demonstrated the potential for heatwave conditions. The analysis compared ambulance attendance on heatwave days with non-heatwave days at an aggregate level. This analysis used heatwave-postcode days as a proxy for person-time risk to calculate incidence rate ratios (IRR).

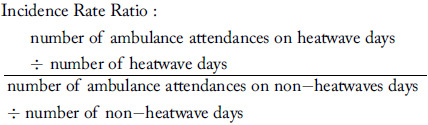




The climate zone boundaries used by the ABCB were matched with postcode correspondences to enable an analysis of heatwave-related ambulance attendance by climate zones. Ambulance attendance by demographic characteristics of age and sex were also performed.

The IRRs and 95% confidence intervals (CI) were constructed using Microsoft Excel (Microsoft Corp.; Redmond, Washington USA).^32^ To address the primary aim, a P value for the main effect was calculated using the Open-Source Statistics for Public Health ‘Compare 2 Rates’ calculator (OpenEpi).^33^ To address the secondary aims, independent IRRs were calculated separately for each stratum, stratifying by age group, sex, remoteness, climate zone, and reason for attendance. Confidence intervals for secondary outcomes were calculated separately for each stratum and were not adjusted for multiplicity, and therefore should be used for hypothesis generation only. Confidence intervals with significant direct effects are marked as significant.

### Ethics

Ethics approval was obtained (Children’s Health Queensland HHS HREC [Brisbane, Queensland, Australia]: LNR/21/QCHQ/72461). Application for release of health information held by QAS for the purposes of research was also obtained.

## Results

### Descriptive Summary

There were 444/450 postcodes that experienced a heatwave and at least one ambulance attendance during the ten years in Queensland. There were 142,584 heatwave-postcode days experienced in Queensland from January 1, 2010 through December 31, 2019. There were 776,850 ambulance attendances across all of the days where a heatwave was experienced, resulting in 648,070 ambulance transports on heatwave days (83.4% of all attendances on heatwave days were transported). Comparatively, there were 1,900,090 ambulance attendances and 1,583,569 transports on non-heatwave days (83.3% of all attendances on non-heatwave days were transported).

### Primary Outcome

The IRR of ambulance attendance was overall 9.3% greater during heatwave days than non-heatwave days (IRR = 1.09; 95% CI, 1.09-1.10; P <.001 - significant).

### Secondary Outcomes

#### Demographics—

Ambulance attendance increased during heatwave days across all age groups with the highest increase of 13% for adults aged 85 years and older (IRR = 1.13; 95% CI, 1.12-1.14 - significant); Table 1. Females and males both experienced increased ambulance attendance during heatwave days accounting for a ten percent and nine percent greater attendance, respectively (IRR = 1.10; 95% CI, 1.10-1.11 - significant and IRR = 1.09; 95% CI, 1.08-1.09 - significant).

**Table 1.** Incidence Rate Ratio of Ambulance Attendance (Heatwave versus Non-Heatwave) for Age Groups and Sex by Rurality (Queensland; 2010-2019)

**Incidence Rate Ratio** **[Unadjusted 95% Confidence Interval]**							
		Major Cities	Inner Regional	Outer Regional	Remote	Very Remote	Total
**Age**	0-14 Years	**1.05 [1.04, 1.06]**	1.00 [0.98, 1.02]	0.97 [0.95, 1.00]	**0.88 [0.83, 0.93]**	1.02 [0.95, 1.09]	**1.04 [1.03, 1.05]**
	15-24 Years	**1.07 [1.06, 1.08]**	**1.04 [1.02, 1.06]**	1.01 [0.99, 1.03]	0.98 [0.94, 1.03]	**1.12 [1.05, 1.19]**	**1.08 [1.07, 1.09]**
	25-34 Years	**1.12 [1.11, 1.13]**	**1.06 [1.04, 1.09]**	**1.04 [1.02, 1.06]**	0.96 [0.91, 1.00]	**1.11 [1.04, 1.19]**	**1.12 [1.11, 1.13]**
	35-44 Years	**1.12 [1.10, 1.13]**	**1.06 [1.04, 1.08]**	1.00 [0.98, 1.03]	0.94 [0.90, 0.99]	1.05 [0.98, 1.12]	**1.11 [1.10, 1.12]**
	45-54 Years	**1.11 [1.10, 1.12]**	**1.07 [1.05, 1.09]**	**1.04 [1.02, 1.06]**	0.97 [0.92, 1.02]	**1.16 [1.09, 1.23]**	**1.11 [1.10, 1.12]**
	55-64 Years	**1.09 [1.07, 1.10]**	**1.06 [1.04, 1.08]**	1.01 [0.99, 1.04]	1.02 [0.96, 1.08]	**1.11 [1.04, 1.18]**	**1.09 [1.08, 1.10]**
	65-74 Years	**1.11 [1.09, 1.12]**	**1.09 [1.07, 1.11]**	1.00 [0.98, 1.02]	1.02 [0.96, 1.09]	0.93 [0.87, 1.00]	**1.11 [1.10, 1.12]**
	75-84 Years	**1.09 [1.08, 1.10]**	**1.09 [1.07, 1.11]**	1.01 [0.99, 1.03]	**1.09 [1.02, 1.17]**	1.07 [0.99, 1.15]	**1.11 [1.10, 1.12]**
	85+ Years	**1.10 [1.09, 1.11]**	**1.08 [1.06, 1.10]**	**1.07 [1.04, 1.10]**	1.01 [0.92, 1.12]	0.92 [0.82, 1.02]	**1.13 [1.12, 1.14]**
**Sex**	Female	**1.09 [1.08, 1.10]**	**1.07 [1.05, 1.08]**	**1.02 [1.01, 1.04]**	0.98 [0.95, 1.02]	**1.13 [1.09, 1.17]**	**1.10 [1.10, 1.11]**
	Male	**1.09 [1.08, 1.10]**	**1.06 [1.04, 1.07]**	1.01 [0.99, 1.02]	**0.96 [0.93, 0.99]**	1.01 [0.97, 1.05]	**1.09 [1.08, 1.09]**
	Indeterminate	1.04 [0.95, 1.14]	0.90 [0.76, 1.09]	0.93 [0.78, 1.10]	1.49 [0.88, 2.51]	1.06 [0.58, 1.91]	1.02 [0.95, 1.10]

Note: Bold values indicate a significant incident rate ratio; Values are rounded to the second decimal place.

#### *G*eographical Area—

**Figure 2.**
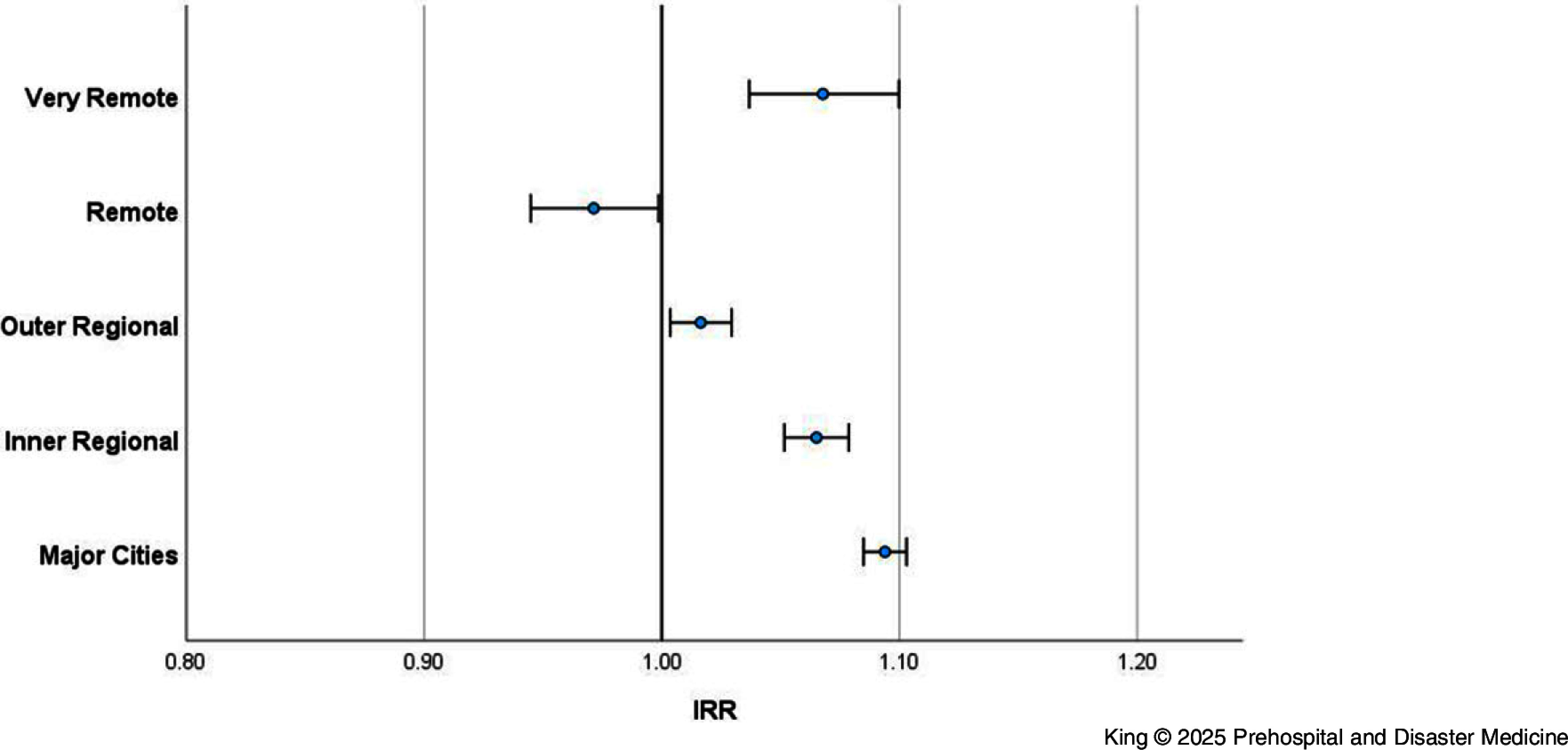
Incidence Rate Ratio of Ambulance Attendance (Heatwave versus Non-Heatwave) by Rurality (Queensland; 2010-2019). Abbreviation: IRR, Incidence Rate Ratio.

The IRR for ambulance attendance differed by rurality. Rates of ambulance attendance were nine percent higher on heatwave compared to non-heatwave days in major cities (IRR = 1.09; 95% CI, 1.08-1.10 - significant); 6.5% higher in inner regional (IRR = 1.07; 95% CI, 1.05-1.08 - significant); 1.6% higher in outer regional (IRR = 1.02; 95% CI, 1.00-1.03 - significant); and 6.7% higher in very remote areas (IRR = 1.07; 95% CI, 1.07-1.10 - significant). A slight protective effect was found in remote locations, although this was not significant (IRR = 0.97; 95% CI, 0.94-1.00); Figure 2.

The rate of ambulance attendance increased significantly on heatwave versus non-heatwave days in major cities across all age groups and in inner regional areas for all age groups, except children aged 0-14 years (Table 1; Supplementary File 2 [available online only] shows more detailed data on age group, sex, and rurality). Generally, rates were also higher (but not significantly) in outer regional, remote, and very remote areas in all age groups, and in some instances, a protective effect was found including for children aged 0-14 years in outer regional areas (IRR = 0.97; 95% CI, 0.95-1.00), remote areas (IRR = 0.83; 95% CI, 0.83-0.93), and for those aged 35-44 years in remote areas (IRR = 0.94; 95% CI, 0.90-0.99); Table 1.

Rates of ambulance attendance during a heatwave (versus non-heatwave) were significantly higher for females in all geographical locations except remote locations. For males, rates were significantly higher on heatwave days in major cities (IRR = 1.09; 95% CI, 1.08-1.10 - significant) and inner regional locations (IRR = 1.06; 95% CI, 1.04-1.07 - significant), and significantly lower in remote areas (IRR = 0.96; 95% CI, 0.93-0.99 - significant); Table 1.

#### Climate Zone—

**Figure 3.**
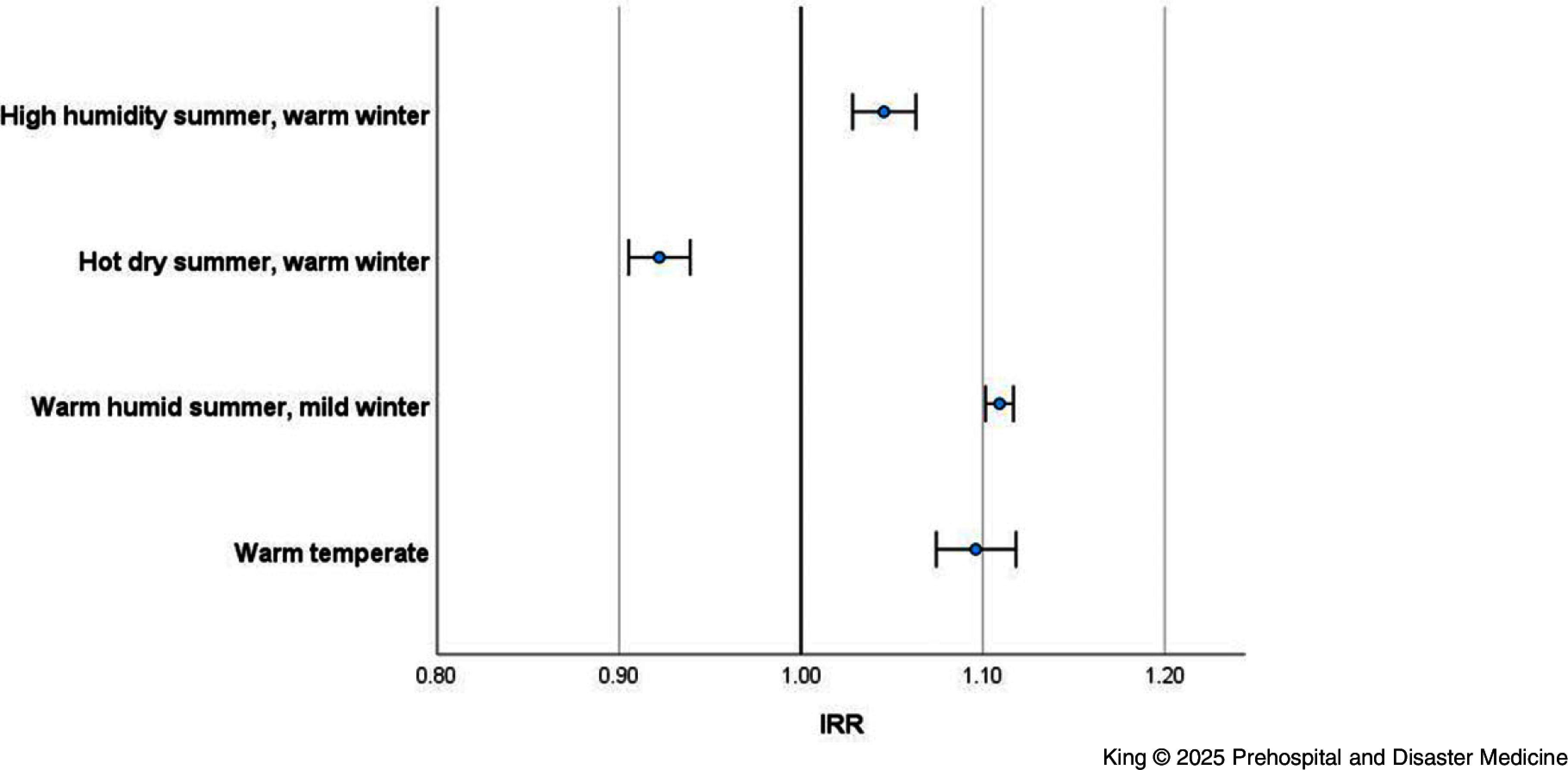
Incidence Rate Ratio of Ambulance Attendance (Heatwave versus Non-Heatwave) by Climate Zone (Queensland; 2010-2019). Abbreviation: IRR, Incidence Rate Ratio.

Exploring whether different climate zones experience a higher risk of ambulance attendance on heatwave days found a protective effect for locations that experience hot dry summers and warm winters (IRR = 0.92; 95% CI, 0.91-0.94), and a small but significant increased risk for the other zones (Figure 3).

#### Reason for Attendance—

Significant increases in rates of ambulance attendance were observed on heatwave days relative to non-heatwave days, for most reasons for attendance. Most profoundly, the rate of attendance increased for heat exposure (IRR = 3.96; 95% CI, 3.81-4.12 - significant), dehydration (IRR = 1.79; 95% CI, 1.74-1.84 - significant), alcohol-drugs (IRR = 1.57; 95% CI, 1.55-1.59 - significant), and sepsis (IRR = 1.46; 95% CI, 1.43-1.50 - significant); Table 2. Heat exposure incorporates conditions such as heat stress, heat stroke, sunburn, and hyperthermia. Supplementary File 3 (available online only) shows IRR by sex and reason for attendance.

**Table 2.** Incidence Rate Ratio of Ambulance Attendance by Reason for Attendance (Queensland; 2010-2019)

Reason for Attendance	**Incidence Rate Ratio** **[Unadjusted 95% Confidence Interval]**
Alcohol/Drugs	**1.57 [1.55, 1.59]**
Allergies/Allergic Reaction	**1.11 [1.08, 1.13]**
Cardiac	**1.09 [1.08, 1.11]**
Cold Exposure	0.56 [0.44, 0.72]
Deceased	**1.09 [1.05, 1.13]**
Deep Vein Thrombosis	0.99 [0.89, 1.09]
Dehydration	**1.79 [1.74, 1.84]**
Endocrine	**1.09 [1.07, 1.12]**
Faint/Dizziness	**1.15 [1.14, 1.17]**
Gastrointestinal	**1.08 [1.07, 1.09]**
Genitourinary	**1.12 [1.10, 1.14]**
Headache/Migraine	**1.14 [1.12, 1.16]**
Heat Exposure	**3.96 [3.81, 4.12]**
Infection (Non-Respiratory)	**1.22 [1.20, 1.24]**
Injuries	**1.07 [1.07, 1.08]**
Mental Health	**1.15 [1.14, 1.16]**
Neurological	**1.04 [1.03, 1.05]**
No Problem Identified	**1.04 [1.02, 1.05]**
Obstetric	**1.13 [1.10, 1.16]**
Other	0.95 [0.94, 0.95]
Respiratory	**1.09 [1.08, 1.10]**
Sepsis	**1.46 [1.43, 1.50]**
Specified Medical	**1.12 [1.11, 1.12]**

Note: Bold values indicate a significant incidence rate ratio; Values are rounded to the second decimal place.

## Discussion

Heatwaves impact ambulance services, and EMS such as QAS play a critical role in responding to climate-related health crises.^34^ In its 2022-2027 strategy, QAS acknowledges that the service will experience challenges and opportunities in responding quickly to complex and compounding system-wide threats related to climate change.^20^ This is likely to compound the already present increase in health service and service demand, as evidenced by experiences in the capital, Brisbane.^35,36^ It is timely to consider the historical impacts of heatwaves on ambulance attendance rates in the whole state to assess the impact and inform preparedness at a detailed macro-level.

In this study, heatwaves greatly increased the rate of ambulance attendance; there were also notable differences by sex, age groups, rurality, and reason for attendance, demonstrating heatwaves dynamic impacts on ambulance work demands in Queensland. Exploring the impacts on EMS service demand is necessary to inform system preparedness, improve vulnerability assessments and risk communication, as well as prompt considerations of work demands, practice improvements, and opportunities for heatwave-specific data collection.

### Understanding Vulnerability

The impact of heatwaves on ambulance attendance varied by rurality, with major cities experiencing the greatest increase in demand on heatwave days in comparison to non-heatwave days. This may be driven by the urban heat island effect, where metropolitan areas experience higher temperatures due to heat absorption and retention by buildings, roads, and other urban infrastructure, reduced vegetation cover, and anthropogenic heat sources.^37,38^ However, it is noted that inner regional and very remote areas also saw notable increases in ambulance attendance in this study, suggesting additional factors may be at play.

The results provide preliminary insights into the reasons individuals require ambulance attendance, including the conditions and/or symptomology to be most aware of during heatwaves. Naturally, there was a significant increase in ambulance attendance for “heat exposure-related conditions” such as heat stress or heat stroke. However, the increased demand also occurred for cardio-respiratory, neurological, and mental health conditions. There were particularly pronounced increases in attendance for dehydration, alcohol and/or drug related events, and sepsis. Thus, EMS personnel may require additional training and resources to manage these heat-related health burdens efficiently, including revised triage protocols and enhanced coordination with hospital emergency departments to maximize system efficiency.^39^ Further work to disentangle the mechanisms by which periods of cumulative excess heat confer these health effects is needed to aid in prevention, tailored risk communication, and to aid in disaster preparedness.

Heatwaves have two mechanisms of impacting human health. High ambient heat may directly cause physiological distress that disturbs human thermoregulation or exacerbates chronic conditions.^11^ There are noted to be major differences in thermoregulatory capacity based on age.^40^ Heatwaves may also cause dehydration which disturbs circulation and tissue perfusion resulting in respiratory, cardiac, or renal failure or may result in thrombosis that can cause stroke or heart attacks.^41^ These mechanisms of effect may, in turn, be moderated or exacerbated by pharmacological, behavioral, or environmental influences. This dual burden of emerging and exacerbating health experiences distinguishes heatwaves from other natural hazard events but also principally highlights that the effects are context-specific.

It is widely acknowledged that heatwaves impact people with pre-existing health conditions,^4^ but there is less recognition of temporal health impairments that are intricately connected to exposure and behaviors in the lead-up to and during heatwaves. There is also the potential that health impairments due to heatwaves can continue to manifest after the heatwave event has passed, as shown in exploring the impact of lag days after events.^10,42–44^ Understanding the interconnection between heat exposure, dehydration, alcohol and drug use, and sepsis is critical to health service planning and associated public service messaging.

### Climatic Conditions, Behaviors, and Tolerance

The EHF uses localized temperature anomaly data, and as such, the characteristics of a heatwave experience are intrinsically connected to the climatic conditions in the region in which they occur. The EHF doesn’t take into account humidity in its calculations, although it is acknowledged that humidity compounds the experience of extreme heat. The inclusion of minimum temperature partially compensates where higher values may be driven by higher humidity. The EHF is also effective in Queensland’s humid locations due to the correlation between temperature and humidity during the heat season.

Three climate zones that experience warm temperate, warm humid, and high humidity summers (respectively) showed higher ambulance attendance rates than the hot dry summer zone. There is the potential that pre-existing high humidity could mask the physiological experience of the temperature anomaly reducing the stimulus for behavioral adaptation. Acclimated residents in the future will need to contend with the continued warming and the limits of human adaptation and physiology. Recent research highlights the thresholds of human adaption capabilities including the upper limits of compensability in conditions of extreme moist heat.^45,46^ Furthermore, disentangling the impact of humidity on heat-related health outcomes is being advocated for and aligns with the need for physiological assessments and multidisciplinary research teams.^47^

The finding that ambulance attendance is reduced in climate zones characterized by hot dry summers and warm winters also suggests there is more to unpack about heatwaves, humidity, and climatic zones independent of and in combination with health service impacts. A potential confounding variable is the proximity and density of health services, population size, and climate zones. Based on this extended exploration, there could be opportunities to develop or adjust adaptive messages.

### Preparedness Based on Service Demand Patterns

A range of service, facility, and community heatwave plans exist and others are currently being developed across Queensland. To date, there is a Queensland Health Heatwave Management Sub-Plan, Department of Education guidelines, and requirements for workplaces to protect workers from heat-related illness as part of risk assessment practices covered under the Work Health and Safety Act 2011.^48–50^ There are also an array of public facing communications and campaigns, in addition to heat health resources, such as Get Ready Queensland, a year-round, all-hazard, resilience-building initiative that includes a feature on heatwaves as a type of extreme weather event that residents should be aware of as part of understanding risk.^51,52^ The establishment of these specific plans as well as more general climate change adaptation plans such as the *Human Health and Wellbeing Climate Change Adaptation Plan for Queensland* and the *Emergency Management Sector Adaptation Plan for Climate Change* highlight the chain of command and delegation of responsibilities that will be activated during events including heatwaves.^53,54^ Service demand planning occurs across the whole of the acute care system including ambulance, emergency department, and hospital admissions. Development and activation of surge plans and wider prehospital system planning can assist in addressing the ten percent increase in service demand noted to be found across the state, in addition to location-specific service surge planning.

Understanding the risk profile is critical to the design of interventions at the structural, individual, or community level. The capacity to implement precautions and monitor consequences requires targeted risk communication at the community and individual levels about vulnerability. However, not all individuals will know their vulnerability and/or have the capacity to instigate precautionary exposure mitigation or employ cooling mechanisms.^8^ Vulnerability mapping to reduce thermal stress, deployment of cool retreats, and communication regarding their availability needs to occur in all heatwaves.^48^

### Changed Work Practices and Demand due to Cascading and Compounding Heatwaves Impacts

Alongside Queensland Health and the Queensland Fire Department, QAS is being proactive in understanding the implications of the changing climate on work demand and practices. This study has highlighted how heatwaves increase service demand, however, it hasn’t explored the paramedics’ perspective on how heatwaves impact their service delivery (including the impact on staff well-being and physiological impacts of personal protective equipment in extreme heat conditions). Paganini, et al highlighted heatwaves increase workload for EMS personnel, who may experience heightened fatigue, worsened by heavy protective gear, prolonged response times, and inadequate vehicle cooling systems.^39^ This suggests the need for EMS-specific protocols that enhance occupational safety during extreme heat events, such as the use of personal cooling garments.^55^

Heatwaves also have the potential to influence infrastructure and thereby service delivery, including through power outages influencing the ease and speed with which services can be provided.^38^ Having surge plans, service training for responding during extreme temperatures, and more generally, having a robust system that can cope with increasing demands reinforces the need for workforce, service, and system resilience and capacity building. The capacity to activate surge plans is influenced by the anticipation of heatwaves through early warning systems. The 2022 *Early Warnings for All* report demonstrates the global need for enhancing capacity to detect hazards as a key action area to ensure warning services can be employed.^56^

## Strengths and Limitations

This study used a comprehensive ten-year data set for the entire state of Queensland to investigate changes in ambulance attendance during heatwaves in comparison to non-heatwave conditions. The analysis allowed for the detailed stratification by demographic, geographic, and medical variables. However, this study reports crude and stratum-specific IRRs, which have not been adjusted for confounding variables nor multiplicity and should be used for hypothesis generation only.

This study utilizes data from ten years that incorporates a change in QAS databases (eARF and dARF). While care was taken to utilize equivalent data from the different databases, nonetheless, there remain differences in key variables that were extracted from the two databases, particularly in relation to reason for attendance. Further, there is no single definitive variable that adequately describes the reason for attendance as required for this research, so a composite variable was constructed through a process of advanced triangulation, guided by medical and prehospital expertise. This variable includes a mixture of symptoms, diseases, and conditions and includes categories with limited interpretability, such as “specified medical,” “no problem detected,” or “other” (where no reason for attendance could be derived from the available variables). It is possible that a reason for attendance could have been described elsewhere in the ambulance record (eg, in the case narrative, which is a free-text variable), but it was not possible to manually review data from the ten-year study period (over 4.2 million records).

A further limitation is that the data used to determine reason for attendance are based on information available at the time of attendance by paramedics at the event and cannot be considered the definitive diagnosis for the condition experienced by the patient. In addition, the recorded reason for attendance may not represent the true cause of the incident. For example, a fall might be recorded as the reason for attendance, but this might have been caused by dehydration that influenced the movement of the individual, and this caused the fall. Reliable and standard coding and categorizing systems are recommended for the future, in alignment with International Classification of Disease coding system.^57^

Another limitation is the use of heatwave postcode days as a proxy for person-time risk, as it assumes the same exposure across demographic and medical comparison groups. While the authors acknowledge this is unlikely to reflect reality, the lack of demographic information associated with BoM data, an obvious virtue of the nature of the data being captured, challenges the capacity to match EHF with demographic data found in other systems. Integration of health system services to capture when current heatwave events are locally being experienced after the transport has occurred might resolve this issue in future research and enhance future explorations of the impact of heatwaves on health systems.

## Conclusion

Heatwaves are expected to become more frequent and intense so understanding the implications of heatwaves on current health system usage in the prehospital setting is important to inform community, service, and system preparedness. Ambulance service demand increases substantially in Queensland during heatwaves. Ensuring the service and prehospital system can adequately respond to these surges in demand, both now and into the future, is essential.

## Supporting information

King et al. supplementary material King et al. supplementary material

## Supplementary Materials

To view supplementary material for this article, please visit https://doi.org/10.1017/S1049023X25101192
