# Supplementary material for: Ambulance Attendance in the State of Queensland, Australia: Exploring the Impacts of Heatwaves Using a Retrospective Population-Based Study
Source: Prehosp Disaster Med. 2025 Jun 25;40(3):147–55. doi: 10.1017/S1049023X25101192 (PMC12237701; doi:10.1017/S1049023X25101192)
Supplement: King et al. supplementary material [file S1049023X25101192sup001.docx]

**Supplementary File 1.** *Dataset and Variable Triangulation Overview: Reason for Ambulance Attendance composite variable calculation*

| **Dataset** | **Time Period** | **Paramedic Assessment of Reason for Transport** | **Secondary Data Options: Reason for Transport** | | **Action if Paramedic Assessment and Secondary Data Options Missing** | **Thematic Grouping of Assessment Variable: Process of Data Triangulation: Dataset specific** |
| --- | --- | --- | --- | --- | --- | --- |
|  |  |  | **Variable with Pre-Existing Categories** | **Patient Complaint Variable** |  |  |
| eARF | 1 Jan 2008-31 Dec 2017 | Final Assessment^a^ | Case Nature – contains 28 pre-specified response categories including injury categories. | Patient Complaint^b^ | Use MPDS variable^c^ | If ‘Final Assessment’ missing or unknown:  Review ‘**Case Nature’**> If no Case Nature information review ‘**Patient Complaint’**>If no useful information in Patient Complaint use ‘**MPDS’** variable. |
| dARF | 1 Jan 2018-31 Dec 2019 | Primary Diagnosis^a^ | Injury Category – contains 15 pre-specified response categories. | Primary Complaint^b^ | Use MPDS variable^c^ | If ‘Primary Diagnosis’ missing or unknown:  Review ‘**Injury Category’** (if entry into this variable ≥1 category coded as injury)>If no Injury Category look in ‘**Primary Complaint’**>If no Primary Complaint information >Look at ‘**MPDS’** variable. |

**Footnotes:** A composite reason for ambulance attendance was created to utilize consistent coding across final assessment, primary diagnosis, and Medical Priority Dispatch System codes. This composite reason is called ‘Attendance Reason.’ The final categories arrived at from this triangulation process includes a mixture of symptoms, diseases, and conditions and some of the final categories utilized might be attributable to a specific but unspecified underlying disease, condition, medication or due to environmental exposure e.g. faint/dizziness.

^a^ First layer of triangulation was to code FA and PD to categories of ‘Attendance Reason.’ Case nature and injury categories use pre-existing variables and these were triangulated to ‘Attendance Reason.’ ^b^ Second layer of triangulation explored patient-described complaint variables when other information was missing and coded to ‘Attendance Reason.’ Final Assessment outlines what the paramedic believes is the patient’s main problem at the time the patient is discharged from his/her care. Primary (Paramedic) Diagnosis as coded by paramedics based on the information presented to them by the patient and their clinical checks but this does not constitute the final medical diagnosis. ^c^ The final layer of triangulation utilized MPDS codes when no other information could be extracted from the records using the other variables. The MPDS variable uses information entered by the ambulance call taker at the time of emergency call and may not reflect the reason for attendance and the need for medical assistance witnessed or determined at the scene.

**Supplementary File 2.** *Incidence rate ratio of ambulance attendance (heatwave vs non-heatwave) for age groups by sex and rurality (Queensland; 2010-19)*

|  |  | **IRR [Unadjusted 95% Confidence Interval]^a^** | | | | |
| --- | --- | --- | --- | --- | --- | --- |
|  |  | **Major Cities** | **Inner Regional** | **Outer Regional** | **Remote** | **Very Remote** |
| **Female** | 0-14 Years | **1.05 [1.04, 1.07]** | 1.01 [0.98, 1.04] | 1.01 [0.97, 1.04] | **0.90 [0.83, 0.98]** | 1.00 [0.9, 1.12] |
|  | 15-24 Years | **1.08 [1.06, 1.09]** | **1.06 [1.04, 1.09]** | 1.02 [1.00, 1.05] | 1.01 [0.94, 1.07] | **1.18 [1.09, 1.28]** |
|  | 25-34 Years | **1.12 [1.10, 1.13]** | **1.06 [1.03, 1.09]** | **1.05 [1.02, 1.07]** | 0.96 [0.90, 1.03] | **1.27 [1.17, 1.38]** |
|  | 35-44 Years | **1.12 [1.10, 1.13]** | **1.08 [1.05, 1.10]** | 1.00 [0.98, 1.03] | 0.98 [0.91, 1.05] | 1.07 [0.97, 1.17] |
|  | 45-54 Years | **1.12 [1.10, 1.13]** | **1.06 [1.03, 1.09]** | **1.06 [1.03, 1.09]** | 1.03 [0.96, 1.11] | **1.26 [1.17, 1.37]** |
|  | 55-64 Years | **1.09 [1.07, 1.11]** | **1.06 [1.03, 1.09]** | 1.00 [0.98, 1.04] | 1.00 [0.91, 1.10] | **1.12 [1.02, 1.23]** |
|  | 65-74 Years | **1.11 [1.09, 1.12]** | **1.11 [1.09, 1.14]** | 1.01 [0.98, 1.04] | 1.00 [0.9, 1.11] | 1.02 [0.92, 1.14] |
|  | 75-84 Years | **1.08 [1.07, 1.10]** | **1.09 [1.06, 1.11]** | 1.01 [0.98, 1.04] | 1.03 [0.93, 1.14] | 1.10 [0.99, 1.23] |
|  | 85+ Years | **1.10 [1.09, 1.12]** | **1.06 [1.03, 1.08]** | **1.07 [1.04, 1.11]** | 0.98 [0.86, 1.12] | 0.95 [0.83, 1.09] |
| **Male** | 0-14 Years | **1.04 [1.03, 1.06]** | 0.99 [0.97, 1.02] | **0.95 [0.92, 0.98]** | **0.86 [0.80, 0.93]** | 1.02 [0.93, 1.11] |
|  | 15-24 Years | **1.07 [1.05, 1.08]** | 1.01 [0.98, 1.04] | 0.99 [0.96, 1.02] | 0.96 [0.90, 1.03] | 1.06 [0.97, 1.16] |
|  | 25-34 Years | **1.12 [1.10, 1.13]** | **1.07 [1.04, 1.10]** | **1.04 [1.01, 1.07]** | 0.95 [0.89, 1.01] | 0.95 [0.87, 1.05] |
|  | 35-44 Years | **1.12 [1.10, 1.13]** | **1.04 [1.01, 1.07]** | 1.00 [0.98, 1.03] | **0.91 [0.85, 0.97]** | 1.03 [0.94, 1.13] |
|  | 45-54 Years | **1.10 [1.08, 1.12]** | **1.08 [1.06, 1.11]** | 1.02 [1.00, 1.05] | **0.92 [0.86, 0.98]** | 1.06 [0.98, 1.15] |
|  | 55-64 Years | **1.08 [1.07, 1.10]** | **1.07 [1.04, 1.09]** | 1.02 [0.99, 1.05] | 1.03 [0.96, 1.11] | 1.09 [1.00, 1.19] |
|  | 65-74 Years | **1.11 [1.09, 1.12]** | **1.08 [1.05, 1.10]** | 1.00 [0.97, 1.02] | 1.03 [0.95, 1.13] | **0.88 [0.80, 0.96]** |
|  | 75-84 Years | **1.09 [1.08, 1.11]** | **1.10 [1.07, 1.12]** | 1.01 [0.99, 1.04] | **1.14 [1.04, 1.25]** | 1.04 [0.94, 1.15] |
|  | 85+ Years | **1.09 [1.08, 1.11]** | **1.10 [1.07, 1.13]** | **1.07 [1.03, 1.11]** | 1.06 [0.91, 1.24] | 0.85 [0.71, 1.02] |

^a^ Bold values indicate a significant incidence rate ratio
Note: Values are rounded to the second decimal place

**Supplementary File 3.** *Incidence rate ratio of ambulance attendance (heatwave vs non-heatwave) by sex and reason for attendance (Queensland; 2010-19)*

| **Reason for Attendance** | **Males** | | **Females** | |
| --- | --- | --- | --- | --- |
|  | **Occurrences on Heatwave Day** | **Incidence Rate Ratio^a^** | **Occurrences on Heatwave Day** | **Incidence Rate Ratio^a^** |
|  |  | **[Unadjusted 95% Confidence Interval]** |  | **[Unadjusted 95% Confidence Interval]** |
| Neurological | 11,402 | **1.03 [1.01, 1.05]** | 10,602 | **1.06 [1.04, 1.08]** |
| Respiratory | 23,155 | **1.09 [1.07, 1.10]** | 23,667 | **1.12 [1.10, 1.13]** |
| Mental Health | 19,940 | **1.16 [1.14, 1.17]** | 26,935 | **1.14 [1.12, 1.15]** |
| Injuries | 62,910 | **1.07 [1.06, 1.08]** | 51,433 | **1.09 [1.08, 1.10]** |
| Cardiac | 22,828 | **1.10 [1.09, 1.12]** | 22,565 | **1.12 [1.10, 1.14]** |
| Heat Exposure | 1,635 | **3.92 [3.73, 4.12]** | 875 | **4.20 [3.93, 4.49]** |
| Cold Exposure | 33 | **0.51 [0.36, 0.72]** | 26 | **0.60 [0.41, 0.89]** |
| Specified Medical | 92,610 | **1.10 [1.09, 1.11]** | 107,125 | **1.13 [1.12, 1.14]** |
| Other | 33,701 | **0.93 [0.92, 0.95]** | 32,269 | **0.95 [0.93, 0.96]** |
|  |  |  |  |  |
| Alcohol/Drugs | 11,013 | **1.63 [1.59, 1.66]** | 9,427 | **1.57 [1.53, 1.60]** |
| Deceased | 1,751 | **1.08 [1.03, 1.13]** | 885 | **1.11 [1.04, 1.18]** |
| Allergies/Allergic Reaction | 2,518 | **1.09 [1.05, 1.14]** | 3,684 | **1.11 [1.07, 1.15]** |
| Endocrine | 4,242 | **1.09 [1.06, 1.13]** | 3,437 | **1.10 [1.06, 1.13]** |
| Genitourinary | 9,610 | **1.14 [1.11, 1.16]** | 9,198 | **1.11 [1.09, 1.13]** |
| Headache/Migraine | 4,305 | **1.10 [1.07, 1.13]** | 7,400 | **1.17 [1.14, 1.19]** |
| Dehydration | 2,505 | **1.82 [1.75, 1.89]** | 2,193 | **1.75 [1.68, 1.82]** |
| Deep Vein Thrombosis | 166 | 0.97 [0.83, 1.13] | 216 | 1.01 [0.88, 1.15] |
| Faint/Dizziness | 7,436 | **1.16 [1.13, 1.19]** | 10,487 | **1.15 [1.13, 1.17]** |
| Gastrointestinal | 15,919 | **1.07 [1.05, 1.09]** | 22,639 | **1.09 [1.07, 1.10]** |
| Infection (non-respiratory) | 7,212 | **1.21 [1.18, 1.24]** | 6,876 | **1.23 [1.20, 1.26]** |
| No problem identified | 14,247 | **1.02 [1.00, 1.03]** | 14,348 | **1.06 [1.04, 1.07]** |
| Sepsis | 3,460 | **1.44 [1.39, 1.49]** | 2,908 | **1.50 [1.45, 1.56]** |

^a^ Bold values indicate significant incidence rate ratios

Note: Values are rounded to the second decimal place
